# Effects of Avenanthramide on the Small Intestinal Damage through Hsp70-NF-κB Signaling in an Ovalbumin-Induced Food Allergy Model

**DOI:** 10.3390/ijms232315229

**Published:** 2022-12-03

**Authors:** Pan Liu, Tianyi Liu, Mingrui Zhang, Ruixia Mo, Weiwei Zhou, Defa Li, Yi Wu

**Affiliations:** State Key Laboratory of Animal Nutrition, College of Animal Science and Technology, China Agricultural University, Beijing 100193, China

**Keywords:** avenanthramide, food allergy, intestinal damage, Hsp70-NF-κB signaling

## Abstract

A food allergy is caused by an abnormal immune reaction and can induce serious intestinal inflammation and tissue damage. Currently, the avoidance of food allergens is still the most effective way to prevent or reduce allergic symptoms, so the development of new strategies to treat allergies is important. Avenanthramide (AVA) is a bioactive polyphenol derived from oats with a wide range of biological activities; however, it is still not clear whether or how AVA alleviates intestinal damage under allergic situations. The aim of this study was to explore the effect of AVA on the small intestinal damage in an ovalbumin (OVA)-induced food allergy model and its mechanism. In experiment 1, 10 mg/kg bw and 20 mg/kg bw doses of AVA both decreased the serum levels of OVA-specific IgE, histamine, and prostaglandin D induced by OVA. The AVA administration relieved inflammation indicated by the lower serum concentrations of pro-inflammatory cytokines including interleukin-1β, IL-6, and tumor necrosis factor-α. The levels of tight junction proteins including Claudin-1, ZO-1, and Occludin in the jejunum were elevated after AVA administration, accompanied by the improved intestinal morphology. Furthermore, AVA elevated the protein expression of heat shock protein 70 (Hsp70) and inhibited the phosphorylation of nuclear factor kappa-B (NF-κB), thus the apoptozole, which a Hsp70 inhibitor, was applied in experiment 2 to assess the contribution of Hsp70-NF-κB signaling to the effects of AVA. In the experiment 2, the inhibition of Hsp70 signaling treatment abolished the beneficial effects of AVA on the small intestinal damage and other allergic symptoms in mice challenged with OVA. Taken together, our results indicated that AVA exerted an intestinal protection role in the OVA-induced allergy, the mechanism of which was partly mediated by the Hsp70-NF-κB signaling.

## 1. Introduction

Food allergy is defined as an immune-mediated pathological reaction towards food antigens, which is a major health concern and epidemic that affects 5% of adults and 8% of infants in the worldwide [1]. Eggs, peanuts, seafood, milk, and wheat are the most common allergens that cause food hypersensitivity [2]. The mucosal immune system in the intestine is hyporesponsive to oral tolerance antigens due to different T cell events, such as the deletion or anergy of reactive T cells and the induction of regulatory T cells [3,4]. A pivotal feature in the pathogenesis of food allergy is the breakdown of oral tolerance to food allergens, generating a T helper 2 cell (Th2) response that is associated with the IgE production and mast cell degranulation [5,6]. Food allergens can lead to damage in the intestinal barrier and increased intestinal permeability [7]. In the case of damaged epithelium, food allergens are located in the subepithelial tissue, offering the chance of a deviated immune response leading to allergy. In addition, the damaged intestine causes food allergens to enter the blood more easily, further exacerbating allergic reactions and intestinal inflammation [8]. To date, avoidance of allergens is the most common way available to avoid food allergy reactions. Hence, the development of effective strategies to treat the disease is of significant importance.

Natural products are characterized by numerous pharmacological activities, unique properties, and structural diversity. Hence, they can regulate a variety of cells owing to their multi-target and multi-function characteristics. Therefore, natural products can be developed as novel treatment strategies for food allergies. Avenanthramide (AVA) is a group of hydroxycinnamoylan thranilate alkaloids unique to oats among cereals, which have a wide range of biological activities that have been verified in animal and human disease models, including antioxidative, anti-inflammatory, and anticarcinogenic properties via multiple molecular mechanisms [9,10]. AVA has three major isoforms (AVA A, B, and C), and the content of AVA C in oat is higher than the others [11]. Research has shown that AVA can effectively resist mast cell-mediated allergic inflammation in vitro and inhibit the activation of nuclear factor kappa-B (NF-κB) in a dose-dependent manner, thereby reducing inflammatory cytokines concentrations [11,12,13,14]. It is interesting to note that AVA shares a very close chemical structure to Tranilast, an anti-inflammatory and antiallergy drug used in the clinic [15,16]. Tranilast is effective in inhibiting the release of proinflammatory cytokines from mast cells and regulating the activation of NF-κB [17]. Hsp70 is one of the representative members of the heat shock proteins (HSPs) family, and some studies have reported that elevated Hsp70 expression inhibits the production of pro-inflammatory cytokines in various cell types via preventing NF-κB activation [18,19,20]. As a prototypical proinflammatory signaling pathway, NF-κB plays an important role in the expression of proinflammatory genes, and in turn, proinflammatory cytokines such as interleukin-1β (IL-1β) could act as the stimulus signal to activate NF-κB to amplify and prolong the inflammatory response [21]. These findings have important implications for exploring the mechanism of antiallergic activity of AVA.

Therefore, the present study aimed to investigate the effects of AVA on food-allergy -induced intestinal damage in mice, and its underlying mechanism. For this purpose, we used a mouse model of food allergy induced by oral administration of ovalbumin (OVA).

## 2. Results

### 2.1. Effects of AVA on the Allergic and Inflammatory Responses

**Figure 1:**
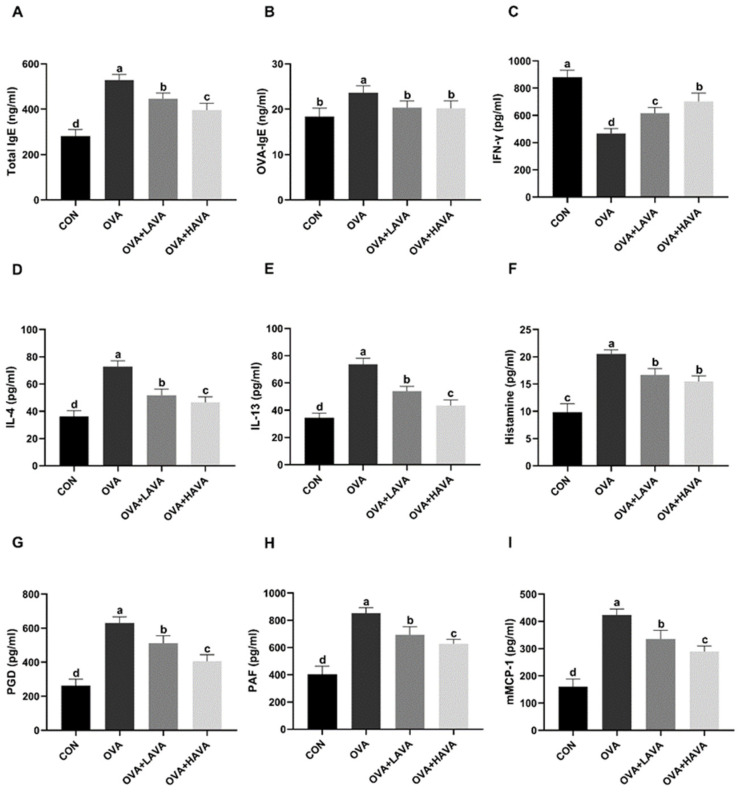
The antiallergic effect of avenanthramide (AVA) in ovalbumin (OVA)-induced food allergy mouse model in experiment 1. (**A**,**B**) Total IgE and OVA-specific IgE (OVA-IgE) in the serum, (**C**–**E**) interferon-γ (INF-γ), interleukin-4 (IL-4), and IL-13 in the serum, (**F**) plasma histamine, (**G**–**I**) prostaglandin D (PGD), platelet activating factor (PAF) and mouse mast cell protease 1 (mMCP-1) in the serum. CON: the control group; OVA: mice orally challenged with OVA; OVA + LAVA: an OVA-challenged group that was oral gavaged with a low dose of 10 mg/kg bw AVA; OVA + HAVA: an OVA-challenged group that was oral gavaged with a high dose of 20 mg/kg bw AVA. Results are presented as means ± SDs (*n* = 8). Bars with no letter in common are significantly different, *p* ≤ 0.05.

In experiment 1, the effects of AVA on the allergic responses were shown in Figure 1. The OVA treatment markedly elevated (*p* ≤ 0.01) the serum levels of OVA-IgE and total IgE in comparison with the CON group. Significantly diminished (*p* ≤ 0.01) productions of OVA-IgE and total IgE were observed in the two doses of AVA supplementations groups compared to the OVA group. Serum levels of Th2 cytokines, including IL-4 and IL-13, were higher (*p* ≤ 0.01) in the OVA group than in the CON group. Supplementations with AVA decreased (*p* ≤ 0.01) the serum levels of IL-4 and IL-13 in comparison to the OVA group. While the serum level of IFN-γ, a marker of T helper 1 cells (Th1), was lower (*p* ≤ 0.01) in the OVA group than the CON group, it was higher (*p* ≤ 0.01) in both AVA supplementation groups compared with the OVA group. Serum concentrations of allergic mediators including PGD, PAF, and mMCP-1 were increased (*p* ≤ 0.01) in the OVA group compared with the CON group, but they were decreased (*p* ≤ 0.01) in the two AVA supplementation groups compared with the OVA group. The plasma histamine was also higher (*p* ≤ 0.01) in the OVA group than that in the CON group, but it was lower (*p* ≤ 0.01) in the two doses of AVA intake groups than that in the OVA group. The high dose of AVA showed better effects on the allergic responses than the low dose, indicated by the increased (*p* ≤ 0.01) IFN-γ as well as the decreased (*p* ≤ 0.01) total IgE, PGD, PAF, IL-4, IL-13, and mMCP-1 in the 20 mg/kg bw dose of AVA intake group compared with the 10 mg/kg bw dose of AVA intake group.

**Figure 2:**
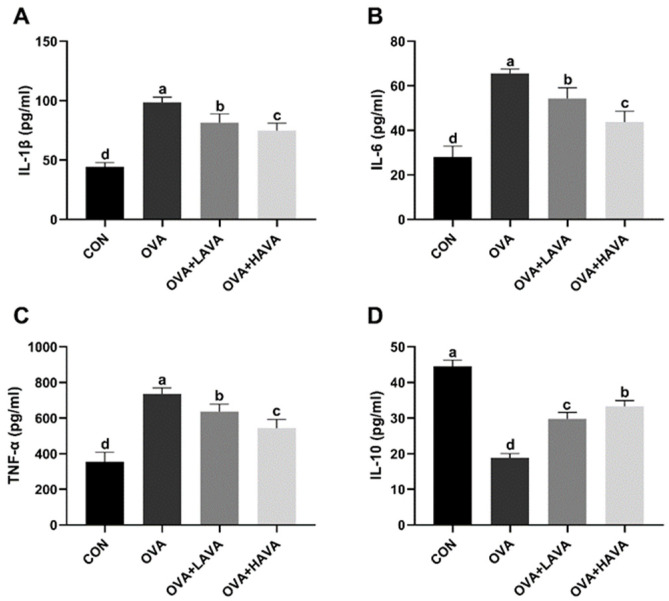
The anti-inflammatory effect of avenanthramide (AVA) in ovalbumin (OVA)-induced food allergy mouse model in experiment 1. (**A**–**C**) Pro-inflammatory cytokines including interleukin-1β (IL-1β), IL-6, and tumor necrosis factor-α (TNF-α) in the serum, (**D**) the anti-inflammatory factor IL-10 in the serum. CON: the control group; OVA: mice orally challenged with OVA; OVA + LAVA: an OVA challenged group that was oral gavaged with a low dose of 10 mg/kg bw AVA; OVA + HAVA: an OVA challenged group that was oral gavaged with a high dose of 20 mg/kg bw AVA. Results are presented as means ± SDs (*n* = 8). Bars with no letter in common are significantly different, *p* ≤ 0.05.

Allergic reactions are often accompanied by inflammatory responses. Serum inflammatory cytokines thus were measured to investigate the inflammatory injury in OVA-induced allergic mice (Figure 2). The OVA treatment increased (*p* ≤ 0.01) serum pro-inflammatory cytokines, including IL-1β, IL-6, and TNF-α compared to the CON group. The two doses of AVA intake groups showed significant attenuation (*p* ≤ 0.01) of serum IL-1β, IL-6, and TNF-α concentrations compared to the OVA group. In contrast, the serum level of anti-inflammatory cytokine IL-10 was decreased (*p* ≤ 0.01) in the OVA group compared with the CON group, but was increased (*p* ≤ 0.01) in the AVA intake groups compared with the OVA group. The IL-1β, IL-6, and TNF-α were lower (*p* ≤ 0.01), but IL-10 was higher (*p* ≤ 0.01) in the 20 mg/kg bw dose of AVA intake group than those in the 10 mg/kg bw dose of AVA intake group. 

### 2.2. Effects of AVA on the Allergic and Inflammatory Responses in the Jejunum

The effects of AVA on the allergic and inflammatory responses in the jejunum of mice were also determined (Table 1). The level of IL-4 in the OVA group was decreased (*p* ≤ 0.01) compared with the CON group but was increased (*p* ≤ 0.01) in both groups supplemented with AVA compared with the OVA group. The concentration of TNF-α in the jejunum in the CON group was lower (*p* ≤ 0.01) than that in the OVA group but was higher (*p* ≤ 0.01) in the OVA group than that in the AVA intake groups. The OVA group showed decreased (*p* ≤ 0.01) IL-10 and TGF-β compared with the CON group, but the AVA intake groups showed elevated (*p* ≤ 0.01) IL-10 level and TGF-β concentration in comparison with the OVA group. Moreover, IL-25, IL-33, slgA, and TSLP were higher (*p* ≤ 0.01) in the CON group than those in the OVA group and higher (*p* ≤ 0.01) in the AVA intake groups than those in the OVA group. Moreover, IL-4 and TNF-α were lower (*p* ≤ 0.01), but IL-25, IL-33, slgA, and TGF-β were higher (*p* ≤ 0.01) in the 20 mg/kg bw dose of AVA intake group than those in the 10 mg/kg bw dose of AVA intake group.

### 2.3. Effects of AVA on the Small Intestinal Damage Induced by OVA

**Figure 3:**
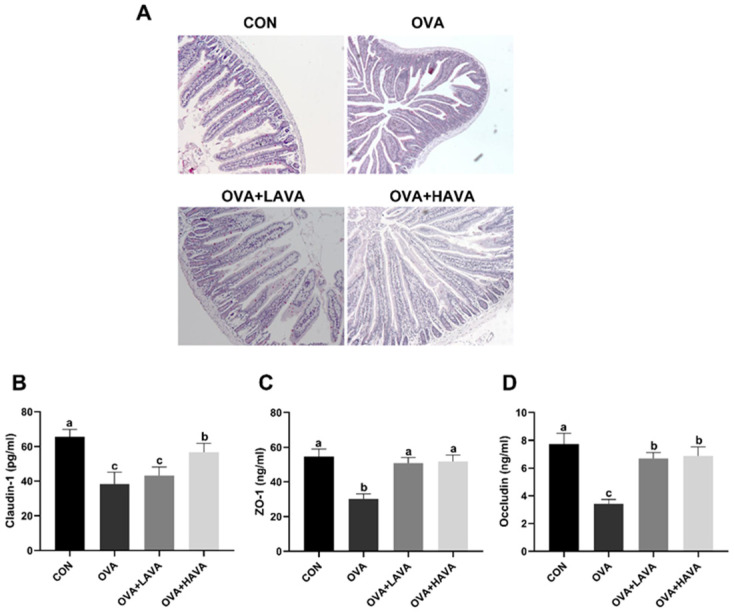
Avenanthramide (AVA) alleviates the intestinal damage induced by ovalbumin (OVA) in experiment 1. (**A**) Intestinal morphology, (**B**–**D**) the expressions of Claudin-1, ZO-1 and Occludin in the jejunum. CON: the control group; OVA: mice orally challenged with OVA; OVA + LAVA: an OVA-challenged group that was oral gavaged with a low dose of 10 mg/kg bw AVA; OVA + HAVA: an OVA-challenged group that was oral gavaged with a high dose of 20 mg/kg bw AVA. Results are presented as means ± SDs (*n* = 9). Bars with no letter in common are significantly different, *p* ≤ 0.05.

As shown in Figure 3A, jejunum morphology of mice in the CON group was normal and intact, whereas the destroyed jejunal tissue morphology was observed in the OVA group. Importantly, mice in the OVA + LAVA and OVA + HAVA groups showed recovered jejunum morphology. Tight junction proteins are closely associated with the intestinal barrier (Figure 3B,C). Consistently, Claudin-1, ZO-1, and Occludin were remarkably lower (*p* ≤ 0.01) in the OVA group than those in the CON group. However, significantly elevated (*p* ≤ 0.01) expressions of Claudin-1, ZO-1, and Occludin were found in the AVA intake groups compared with the OVA group, and the jejunum level of Claudin-1 were higher (*p* ≤ 0.01) in the OVA + HAVA group than the OVA + LAVA group. 

### 2.4. Effect of AVA on the Intestinal Hsp70-NF-κB Signaling

**Figure 4:**
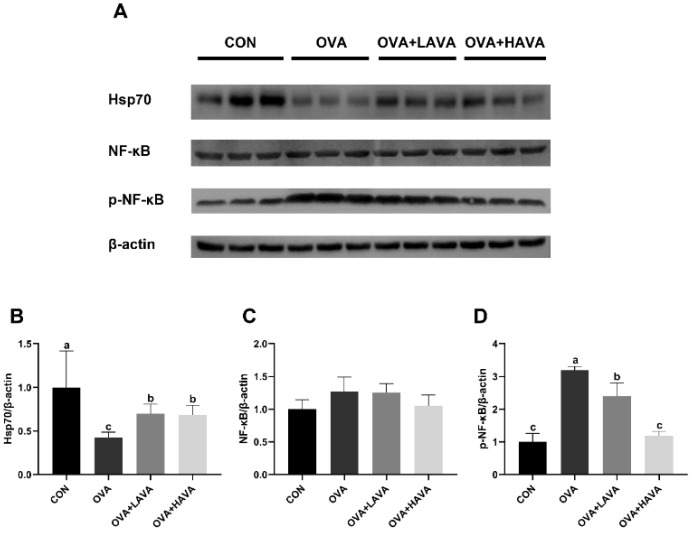
Effects of avenanthramide (AVA) on the Hsp70-NF-κB signaling in the jejunum of mice induced by ovalbumin (OVA) in experiment 1. (**A**) Protein expressions of heat shock protein 70 (Hsp70), nuclear factor kappa-B (NF-κB), phosphorylated-NF-κB (p-NF-κB), and β-actin in the jejunum, (**B**–**D**) Western blot quantitative data of Hsp70, NF-κB, and p-NF-κB in the jejunum. CON: the control group; OVA: mice orally challenged with OVA; OVA + LAVA: an OVA-challenged group that was oral gavaged with a low dose of 10 mg/kg bw AVA; OVA + HAVA: an OVA-challenged group that was oral gavaged with a high dose of 20 mg/kg bw AVA. Results are presented as means ± SDs (*n* = 3). Bars with no letter in common are significantly different, *p* ≤ 0.05.

We investigated the effect of AVA on intestinal Hsp70-NF-κB signaling, and the results were shown in Figure 4. In the experiment 1, the protein expression of Hsp70 in the jejunum was decreased (*p* ≤ 0.01) in the OVA group relative to the CON group. In addition, jejunum Hsp70 expression was significantly increased (*p* ≤ 0.01) in the AVA intake groups compared with the OVA group. Correspondingly, the phosphorylated-NF-κB (p-NF-κB) expression was higher (*p* ≤ 0.01) in the OVA group than that in the CON group, while AVA supplementation significantly blocked the effect raised by OVA challenge, and the OVA + HAVA group showed lower (*p* ≤ 0.01) expression of p-NF-κB than the OVA + LAVA group. Taken together, these results indicated that AVA alleviated the intestinal damage induced by OVA exposure via mediating the Hsp70-NF-κB signaling. 

### 2.5. Effect of Hsp70 Activity Inhibition on the Function of AVA in the Allergic Symptoms and the Small Intestinal Damage Induced by OVA

**Figure 5:**
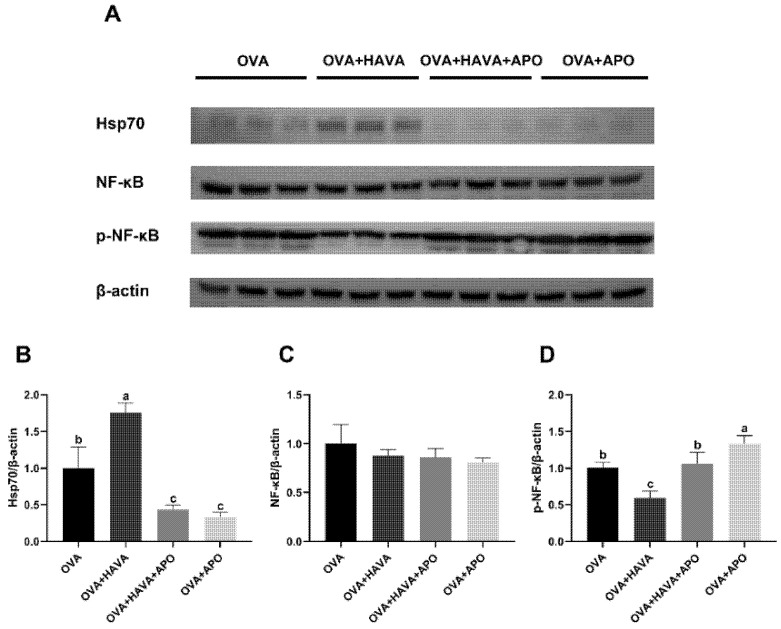
The Hsp70-NF-κB signaling in the jejunum of mice in experiment 2. (**A**) Protein expressions of heat shock protein 70 (Hsp70), nuclear factor kappa-B (NF-κB), phosphorylated-NF-κB (p-NF-κB), and β-actin in the jejunum, (**B**–**D**) Western blot quantitative data of Hsp70, NF-κB and p-NF-κB in the jejunum. OVA: mice orally challenged with OVA; OVA + HAVA: an OVA challenged group that was oral gavaged with a high dose of 20 mg/kg bw avenanthramide (AVA); OVA + HAVA + APO: the OVA + HAVA group coupled with apoptozole (APO); OVA + APO: the OVA group coupled with APO. Results are presented as means ± SDs (*n* = 3). Bars with no letter in common are significantly different, *p* ≤ 0.05.

The results showed above suggested a critical involvement of Hsp70 in the AVA-mediated beneficial effects on intestine. We further hypothesized that Hsp70-NF-κB signaling is responsible for the alleviation of allergic inflammation of AVA. Hence, experiment 2 was conducted to assess the contribution of Hsp70-NF-κB signaling to the effects of AVA; thus, apoptozole, which acts as a Hsp70 inhibitor, was used. As shown in Figure 5, the jejunum Hsp70 expression was increased (*p* ≤ 0.01) in the OVA + HAVA group, while it was inhibited (*p* ≤ 0.01) in the OVA + APO and the OVA + HAVA + APO groups when compared with the OVA group. Relatively, by comparison with the OVA group, the OVA + HAVA group showed an attenuated (*p* ≤ 0.01) level of jejunum p-NF-κB. The p-NF-κB expression in the jejunum was higher (*p* ≤ 0.01) in the OVA + HAVA + APO group than that in the OVA + HAVA group, but was lower (*p* ≤ 0.01) in the OVA + HAVA + APO group than that in the OVA + APO group. 

**Figure 6:**
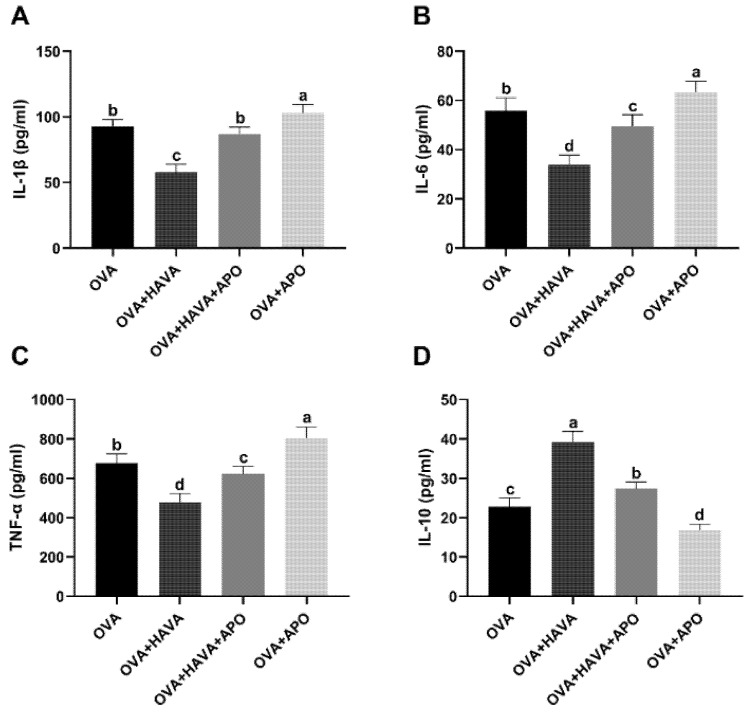
The heat shock protein 70 (Hsp70) inhibition weakened the anti-inflammatory effect of avenanthramide (AVA) in ovalbumin (OVA)-induced food allergy mouse model in experiment 2. (**A**–**C**) Pro-inflammatory cytokines including interleukin-1β (IL-1β), IL-6 and tumor necrosis factor-α (TNF-α) in the serum, (**D**) the anti-inflammatory factor IL-10 in the serum. OVA: mice orally challenged with OVA; OVA + HAVA: an OVA challenged group that was oral gavaged with a high dose of 20 mg/kg bw avenanthramide (AVA); OVA + HAVA + APO: the OVA + HAVA group coupled with apoptozole (APO); OVA + APO: the OVA group coupled with APO. Results are presented as means ± SDs (*n* = 8). Bars with no letter in common are significantly different, *p* ≤ 0.05.

The effect of Hsp70 inhibition on the anti-inflammatory effect of AVA was shown in Figure 6. The OVA + HAVA group showed decreased (*p* ≤ 0.01) serum levels of IL-1β, IL-6, and TNF-α and an increased (*p* ≤ 0.01) serum IL-10 level compared to the OVA group and the OVA + HAVA + APO group. In addition, when compared to the OVA group and the OVA + APO group, the OVA + HAVA + APO group showed declined (*p* ≤ 0.01) serum levels of IL-1β, IL-6, and TNF-α, along with the increased (*p* ≤ 0.01) serum IL-10 level. 

**Figure 7:**
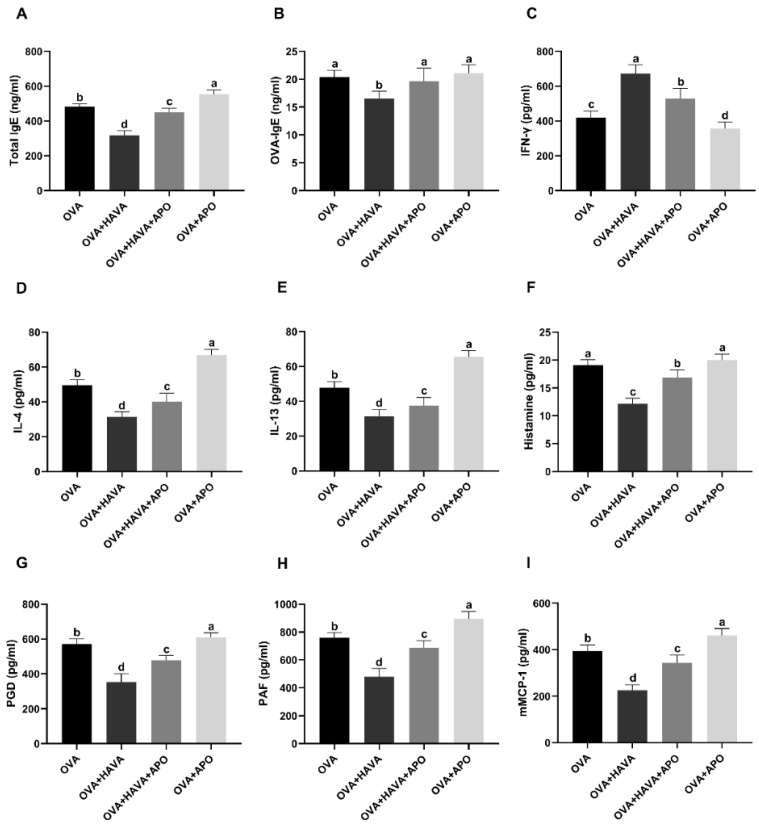
The heat shock protein 70 (Hsp70) inhibition weakened the antiallergic effect of avenanthramide (AVA) in ovalbumin (OVA)-induced food allergy mouse model in experiment 2. (**A**,**B**) Total IgE and OVA-specific IgE (OVA-IgE) in the serum, (**C**–**E**) interferon-γ (INF-γ), interleukin-4 (IL-4), and IL-13 in the serum, (**F**) plasma histamine, (**G**–**I**) prostaglandin D (PGD), platelet factor (PAF), and mouse mast cell protease 1 (mMCP-1) in the serum. OVA: mice orally challenged with OVA; OVA + HAVA: an OVA-challenged group that was oral gavaged with a high dose of 20 mg/kg bw avenanthramide (AVA); OVA + HAVA + APO: the OVA + HAVA group coupled with apoptozole (APO); OVA + APO: the OVA group coupled with APO. Results are presented as means ± SDs (*n* = 8). Bars with no letter in common are significantly different, *p* ≤ 0.05.

The effect of Hsp70 inhibition on the antiallergic effect of AVA was shown in Figure 7. Serum IL-4 and IL-13 levels were decreased (*p* ≤ 0.01) in the OVA + HAVA group in comparison with the OVA group and the OVA + HAVA + APO group, while the serum IFN-γ level was elevated (*p* ≤ 0.01) in the OVA + HAVA group when compared with the OVA group and the OVA + HAVA + APO group. The concentrations of serum total IgE and OVA-IgE were lower (*p* ≤ 0.01) in the OVA + HAVA group than the OVA group and the OVA + HAVA + APO group. Moreover, the levels of PGD, PAF, and mMCP-1 in serum and histamine in plasma were lower (*p* ≤ 0.01) in the OVA + HAVA group than the OVA group and the OVA + HAVA + APO group. In addition, concentrations of IL-4, IL-13, total IgE, PGD, PAF, mMCP-1, and histamine were lower (*p* ≤ 0.01) in the OVA + HAVA + APO group than the OVA group and the OVA + APO group, while the level of IFN-γ was higher (*p* ≤ 0.01) in the OVA + HAVA + APO group than the OVA group and the OVA + APO group. 

Allergic and inflammatory responses under Hsp70 inhibition were also assessed in the jejunum in OVA-induced allergic mice (Table 2). Consistently, compared with the OVA group and the OVA + HAVA + APO group, levels of IL-4 and TNF-α in the jejunum were decreased (*p* ≤ 0.01) in the OVA + HAVA group, while concentrations of IL-10 and TGF-β were increased (*p* ≤ 0.01) in the OVA + HAVA group. The concentrations of IL-25, IL-33 and sIgA in the jejunum were higher (*p* ≤ 0.01) in the OVA + HAVA group than the OVA group and the OVA + HAVA + APO group. Additionally, the levels of IL-4 and TNF-α in the jejunum were lower (*p* ≤ 0.01) in the OVA + HAVA + APO group than the OVA group and the OVA + APO group, while the concentrations of IL-10, TGF-β, IL-25, IL-33, and sIgA in the jejunum were higher (*p* ≤ 0.01) in the OVA + HAVA + APO group than the OVA group and the OVA + APO group. The concentrations of TSLP in the jejunum were not different (*p* > 0.05) among the four groups in experiment 2. 

**Figure 8:**
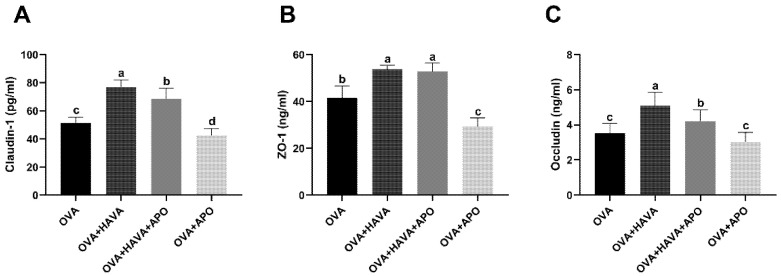
The concentrations of tight junction proteins of jejunum in experiment 2. (**A**–**C**) The concentrations of Claudin-1, ZO-1, and Occludin in the jejunum. OVA: mice orally challenged with OVA; OVA + HAVA: an OVA-challenged group that was oral gavaged with a high dose of 20 mg/kg bw avenanthramide (AVA); OVA + HAVA + APO: the OVA + HAVA group coupled with apoptozole (APO); OVA + APO: the OVA group coupled with APO. Results are presented as means ± SDs (*n* = 9). Bars with no letter in common are significantly different, *p* ≤ 0.05.

The results of the effect of intestinal Hsp70 activity inhibition on the small intestinal damage induced by OVA were shown in Figure 8. Correspondingly, expressions of tight junction proteins, including Claudin-1 and Occludin, were remarkably higher (*p* ≤ 0.01) in mice from the OVA + HAVA group than those in the OVA group and the OVA + HAVA + APO group. In addition, significantly elevated (*p* ≤ 0.01) expressions of Claudin-1, ZO-1, and Occludin were found in the OVA + HAVA + APO group compared to the OVA group and the OVA + APO group. 

Taken together, these results indicated that the effects of AVA may be partly dependent on the Hsp70-NF-κB signaling.

## 3. Discussion

There is currently no definitive treatment for food allergies. Careful allergen avoidance is considered to be an effective form of prevention, but avoidance is not equivalent to a true treatment [22]. Thus, new strategies for treating food allergy development are important. AVA has been shown to have antioxidant activity, anti-inflammatory and anticarcinogenic properties, and in addition, the epidermal antiallergy effect of AVA has also been confirmed [23]. The BALB/c mice have commonly been used for allergy models and have displayed hyperresponsiveness in response to OVA sensitization [24]. In this study, we evaluated the effectiveness of AVA on intestinal damage in food allergy model mice induced by OVA and explored its potential mechanism. All of our results suggested that AVA exerted an intestinal protection role in food allergy model mice induced by OVA, and these beneficial effects were partly due to the promoted intestinal Hsp70 expression and the inhibited activation of NF-κB.

Allergic inflammation refers to the inflammation produced in sensitized subjects after exposure to specific allergens, which consists of the early and the late phase [25]. During the early phase reaction that can occur within minutes of allergen exposure, the produced antigen-IgE mediates activation and degranulation of mast cells which are known as conductor cells in allergic inflammation, leading to the immediate release of a variety of preformed mediators (e.g., histamine, tryptase) as well as newly formed mediators (e.g., PAF, PGD), and the later production of many pro-inflammatory cytokines such as IL-6 and TNF-α. Concomitantly, the accumulation and activation of Th2, eosinophils, and basophils in the inflamed sites take place, causing the onset of the late phase that usually occurs several hours after the early phase. Mediators that trigger the late-phase reaction are considered to come from resident mast cells. The allergic inflammation process caused by prolonged or repetitive exposure to allergic stimulus may eventually turn chronic [26,27,28]. Histamine has an effect on inducing vasodilation, increasing vascular permeability, and triggering edema formation [29]. PAF is an endogenous phospholipid which acts as one of the primary mediators in allergic and inflammatory processes [30]. PGD is the major prostanoid secreted by activated mast cells and also has been corroborated to be implicated in allergic diseases in several studies [31,32]. In this study, two different doses of AVA both decreased levels of plasma histamine as well as serum PAF, PGD, IL-6, and TNF-α, which suggested that AVA relived anaphylactic symptoms in mice. The level of mMCP-1 is a key indicator for detecting mastocyte degranulation. In our study, the level of serum mMCP-1 was significantly reduced in the low and high doses of AVA groups, which illustrates that mastocyte degranulation was alleviated by AVA, and then relieved allergenicity. 

The IFN-γ secreted from Th1 can inhibit IgE production [33]. However, Th2 cytokines such as IL-4, IL-5, and IL-13 are thought to promote allergic reactions including eosinophil infiltration and IgE production, which induce Th2-dependent antibodies [34] and systemic anaphylaxis [35]. In this study, compared with serum from OVA challenge group, serum from AVA supplementation groups showed significantly reduced levels of OVA-IgE and total IgE. Moreover, low and high doses of AVA both upregulated the Th1 cytokine (IFN-γ) but downregulated the Th2-related cytokines, including IL-4 and IL-13, which indicated that AVA may have regulated the Th1/Th2 imbalance by weakening the Th2 immune response and promoting the Th1 immune response, thereby suppressing the OVA-induced allergic reactions. 

Oral exposure to the allergen induces intestinal histological changes that are associated with the intestinal chronic inflammation state, and research has demonstrated a close relationship between intestinal damage and food allergy [36,37]. Tight junction proteins, including Claudin-1, ZO-1, and Occludin, work to build the permeability barrier of epithelia and, under inflammatory conditions, the membrane permeability increases [38]. In our study, the intestinal morphology damage was identified in the OVA-induced allergic reaction animals, whereas it was markedly improved by AVA consumption. In addition, as a result of experiment 1 in this study, the levels of Claudin-1, ZO-1, and Occludin in the jejunum of high-dose AVA-treated mice were all remarkably increased compared with the OVA group, which suggested that AVA had protection effects on reducing of epithelial permeability and preserving epithelial barrier function. 

Research about the immune effect of AVA on other allergic conditions report increased expression of anti-inflammatory cytokines and decreased expression of pro-inflammatory cytokines in tissue with allergic condition, such as skin in atopic dermatitis [23,39]. Anti-inflammatory cytokines IL-10 and TGF-β, which were produced by regulatory T cells, are crucially involved in the maintenance of gut mucosal homeostasis via controlling Th2 responses, and thus suppress the allergic effector response [6,40]. From our results, the levels of IL-10 and TGF-β were significantly decreased in the OVA group, while they were markedly increased in both doses of AVA-treated groups. IgA is an important mucosal antibody with regulatory and protective functions, and TGF-β in intestinal different cells promotes IgA synthesis and maintains a tolerogenic microenvironment [41,42]. In addition, studies have indicated that the upregulation of TGF-β and the inhibitory effect of TNF-α at the intestine of food allergy animals may prevent an impaired intestinal barrier function [43,44]. These reports were similar to our study results. Stressed or damaged intestinal epithelial cells secrete the cytokines IL-25, IL-33, and TSLP, which were also collectively referred to as alarmins, to promote a Th2 cytokine response and amplify food allergic reactions [45]. In our OVA-induced animals, levels of IL-25, IL-33, and TSLP in the small intestine were indeed significantly decreased. Moreover, supplementation of AVA significantly increased the jejunum levels of IL-25, IL-33, and TSLP and prevented histopathological alteration in the intestinal tissue of OVA-induced animals. Our results are in accordance with the protective effect of AVA on the chronic inflammatory process associated with allergic diseases [13,23,39]. Taken together, these results showed that OVA treatment induced intestinal damage, whereas AVA relieved the process with pro-inflammatory cytokine expressing repression and epithelial barrier preservation.

Heat shock proteins (HSPs) are a class of highly conserved proteins from microbes to mammals and play a crucial role in maintaining protein homeostasis by blocking protein misfolding and assisting assembly [20]. In addition to these typical features, HSP administration has been found to prevent or arrest inflammatory damage in experimental disease models [46]. Hsp70 is the best known and most abundant of all HSPs, and several studies have demonstrated that Hsp70 attenuated inflammatory responses both in vivo and in vitro. For instance, the Hsp70-deficient mice showed elevated mortality in comparison to the wild-type mice in a septic shock model [47], and Hsp70 impeded LPS-induced NF-kB activation and pro-inflammatory cytokines production [48]. The role of NF-κB in regulating the expression of pro-inflammatory genes contributing to chronic or acute inflammation has been extensively reviewed. NF-κB proteins normally exist as an inactive state. Upon exposure to stimuli, the NF-κB complex, predominantly p50/p65, is phosphorylated and translocated into the nucleus, inducing pro-inflammatory genes expression by binding to their promoter sites [49,50]. Therefore, we hypothesized that Hsp70-NF-κB might be involved in the anti-inflammatory function of AVA. Indeed, in experiment 1, the protein expression of Hsp70 in the jejunum was higher in the low and high doses of AVA groups than in the OVA group, which subsequently led to inhibited activation of NF-κB and declined release of pro-inflammatory cytokines. It is worth mentioning that there was a positive feedback regulatory mechanism between IL-1 and histamine. IL-1 was capable of inducing mast cells and basophils to release histamines, and in turn the histamine was also able to promote IL-1 production [51,52]. 

In our study, IL-1β was decreased in the AVA treatments, which was along with downregulated histamine. Studies have shown that the intestinal epithelial Hsp70 plays a pivotal role in maintaining intestinal epithelial cells and protecting mucosal integrity [53,54]; this may be the reason for the decrease in jejunal Hsp70 in mice in the OVA group compared with the control group. These results indicated that AVA might improve allergy-induced intestinal injury by Hsp70-NF-κB signaling. In order to further explore the role of Hsp70 in AVA alleviating the allergic-inflammation response, OVA-induced food allergy mice in experiment 2 were treated with both a Hsp70 inhibitor and a high dose of AVA. Apoptozole, the Hsp70 inhibitor, was shown to block the ATPase activity of Hsp70 by binding to its ATPase domain without affecting Hsp70 expression in HeLa cells in vitro [55]. However, we found that the expression of Hsp70 in the jejunum in mice was reduced after apoptozole exposure in this study, which may be due to differences in the tissues and cellular levels. The down-regulated and inhibited Hsp70 resulted in an increase in NF-κB activation in experiment 2. Additionally, correspondingly, we found that the antagonizing of intestinal Hsp70 by apoptozole weakened the antiallergic effects of AVA. These results suggested that AVA exerted its function partly due to the promoted intestinal Hsp70 expression and the inhibited activation of NF-κB. However, in our study, the OVA + HAVA + APO group did not completely eliminate the effects of AVA; therefore, we speculate that AVA not only exerts its effects through Hsp70-NF-κB signaling, and other mechanisms remain to be explored.

## 4. Materials and Methods

### 4.1. Animal Experiments

All experiments were performed according to protocols approved by the Institutional Animal Care and Use Committee of China Agricultural University (permit no. AW31402202-1-4). BALB/c male mice (6 wk old) were used in all experiments, and before experiments were acclimatized for 7 days. Mice were housed at 22 °C with relative humidity of 55 ± 5% and a 12 h light/dark cycle. Mice were offered water and food ad libitum. 

Experiment 1 was conducted to examine the effects of AVA and its concentration on the small intestine in the OVA-induced allergy. Mice were allocated to four groups (*n* = 10): the control (CON) group, OVA group, OVA group with a low dose of 10 mg/kg bw AVA (OVA + LAVA), and OVA group with a high dose of 20 mg/kg bw AVA (OVA + HAVA). On days 0–38, mice in the OVA + LAVA and OVA + HAVA groups were gavaged daily with AVA in saline (Sigma-Aldrich, Madrid, Spain; purity ≥ 98.0%) [11]. On sensitization or challenge days, the AVA-gavaged treatment was performed 1 h after the sensitization or challenge, while mice in the CON group were gavaged daily with saline at the same point. Mice in the OVA, OVA + LAVA, and OVA + HAVA groups were sensitized with 200 μL saline containing 50 μg OVA (Sigma-Aldrich, Madrid, Spain) and 1 mg Alum adjuvant (Thermo Fisher, Waltham, MA, USA) by intraperitoneal injection on day 0 and 14. On days 28–38, mice in those groups were orally challenged with 50 mg OVA every 2 days, for a total of six times. Mice in the CON group were subjected to intraperitoneal sensitization and oral gavage challenge with saline solution.

Experiment 2 was conducted to evaluate the function of Hsp70-NF-κB signaling in the effects of AVA on the small intestinal damage induced by OVA. Mice were allocated to four groups (*n* = 10): OVA group, OVA + HAVA group, OVA + HAVA group coupled with apoptozole—a Hsp70 inhibitor (OVA + HAVA + APO; Selleck, Houston, TX, USA), and OVA group coupled with apoptozole (OVA + APO). Mice in all groups were sensitized and challenged as described in the experiment 1. Besides, on days 0–38, mice in the OVA + HAVA and the OVA + HAVA + APO groups were gavaged daily with AVA (20 mg/kg bw). On sensitization or challenge days, the AVA treatment was performed 1 h after the sensitization or challenge, and mice in the OVA group and the OVA + APO groups were gavaged daily with saline at the same point. In addition, on days 0–38, mice in the OVA + HAVA + APO and the OVA + APO groups were intraperitoneally injected with APO prepared in 20 ul DMSO (4 mg/kg bw) every other day [55]. APO was administered 1 h before the sensitization or challenge, while mice in the OVA and OVA + HAVA groups were intraperitoneally injected with DMSO at this point. 

At the end of these experiments, mice were fasted for 12 h and then euthanized using a compressed source of CO_2_ gas. Serum was collected by centrifugation from whole blood sample at 4000 g for 30 min at a room temperature. Plasma was obtained by centrifugation from whole blood sample collected in EDTA K2 at 4000 g for 10 min at 4 °C. The jejunum tissue was stored in formalin for 24 h and embedded in paraffin and sectioned. Another part of jejunum tissue was collected and kept in liquid nitrogen until analysis. In both experiments, we collected 8 serum samples and 9 jejunal tissue samples from each group for ELISA analysis.

### 4.2. Evaluation of Immunoglobulins and Cytokines

The OVA-specific IgE (OVA-IgE), total IgE and secretory IgA (slgA) were determined using the commercial ELISA kits (Bethyl Laboratories, Inc. Montgomery, TX, USA). Cytokines such as IL-1β, IL-4, IL-6, IL-10, IL-13, IL-25, IL-33, tumor necrosis factor-α (TNF-α), interferon-γ (IFN-γ), transforming growth factor-β (TGF-β), and thymicstromal lymphopoietin (TSLP) were also measured using the commercial ELISA kits (eBioscience, San Diego, CA, USA). The active mediators, such as prostaglandin D (PGD), platelet-activating factor (PAF), mouse mast cell protease 1 (mMCP-1), and histamine were determined using the commercial ELISA kits (Abcam, Cambridge, UK). The levels of intestinal barrier proteins, including Claudin-1, ZO-1 and Occludin, were measured by ELISA kits (MyBioSource, San Diego, CA, USA). 

The OVA-specific IgE, total IgE, IL-1β, IL-4, IL-6, IL-10, IL-13, TNF-α, IFN-γ, PGD, PAF, and mMCP-1 were detected in serum. The histamine was detected in plasma. Additionally, the TNF-α, TGF-β, IL-4, IL-10, IL-25, IL-33, sIgA, and TSLP were detected from the jejunum tissue. The Claudin-1, ZO-1, and Occludin were also detected from the jejunum tissue.

### 4.3. Western Blot

The primary antibody Hsp70 (1:1000) was purchased from Abcam (Abcam, Cambridge, UK). Primary antibodies including β-actin (1:1000), NF-κB p65 (1:1000), and phosphorylated (p)-NF-κB p65 (1:1000) were purchased from Cell Signaling Technology, Inc. (CST, Danvers, Massachusetts, USA). 

Proteins were extracted from the jejunum tissue. Briefly, the jejunum sections were lysed in RIPA buffer with the protease inhibitor on ice, the tissue homogenate was subsequently centrifuged at 15,000 r/min for 10 min at 4 °C, the attainable supernatant was the whole protein extract. Gel electrophoresis was then performed, and the proteins were transferred to polyvinylidene fluoride membranes by electrotransfer. After being blocked in 5% skim milk for an hour, the membrane was incubated with primary antibody overnight at 4 °C, and then with secondary antibody for an hour at room temperature. Finally, the membranes were incubated in ECL reagents and scanned by Gel Image system [56]. 

### 4.4. Histological Analysis

The jejunum tissue was stored in formalin for 24 h and embedded in paraffin and sectioned. Finally, the tissue was stained by Periodic acid–Schiff and the small intestine was checked for damage under microscope.

### 4.5. Statistical Analysis

Data were analyzed by one-way ANOVA analysis and the GLM program of SAS 9.2 (SAS Institute Inc., Cary, NC, USA) in a completely randomized design. A *p* value of *p* ≤ 0.05 was considered statistically significant and 0.05 < *p* ≤ 0.10 was indicative of a differential trend. Data are expressed as the means ± SDs.

## 5. Conclusions

In this study, our results demonstrated that AVA significantly reduced pro-inflammatory cytokine production as well as allergic mediator release in an OVA-induced food allergy model, thus attenuating the small intestinal damage caused by OVA. Moreover, our results revealed that AVA exerted its function partly due to the promoted intestinal Hsp70 expression, which has been confirmed to be capable of inhibiting NF-κB activation. Of course, other mechanisms by which AVA plays a beneficial role remain to be explored. Our study provides a new strategy for alleviating intestinal damage in the case of food allergies.

## Figures and Tables

**Table 1:** Cytokines in the jejunum in experiment 1.

Variable	Control	OVA	OVA + LAVA	OVA + HAVA	*p* Value
IL-4 (pg/mL)	24.87 ± 4.10 ^d^	48.67 ± 3.82 ^a^	44.24 ± 3.80 ^b^	32.76 ± 2.51 ^c^	<0.01
TNF-α (pg/mL)	357.7 ± 36.11 ^d^	686.7 ± 46.93 ^a^	567.3 ± 24.48 ^b^	505.4 ± 37.31 ^c^	<0.01
IL-10 (pg/mL)	40.58 ± 1.78 ^a^	24.15 ± 2.46 ^c^	31.09 ± 2.43 ^b^	33.01 ± 2.25 ^b^	<0.01
IL-25 (pg/mL)	73.09 ± 4.02 ^a^	44.72 ± 3.87 ^d^	52.82 ± 5.24 ^c^	64.42 ± 6.07 ^b^	<0.01
IL-33 (pg/mL)	194.9 ± 9.16 ^a^	100.6 ± 11.46 ^d^	120.5 ± 7.12 ^c^	157.6 ± 16.28 ^b^	<0.01
slgA (ng/mL)	3533 ± 175.3 ^a^	1804 ± 204.9 ^d^	2504 ± 263.7 ^c^	2934 ± 179.6 ^b^	<0.01
TSLP (pg/mL)	41.42 ± 3.65 ^a^	23.03 ± 4.21 ^c^	33.20 ± 3.30 ^b^	36.50 ± 3.54 ^b^	<0.01
TGF-β (pg/mL)	656.9 ± 98.91 ^a^	233.1 ± 55.31 ^d^	407.8 ± 44.22 ^c^	538.8 ± 41.11 ^b^	<0.01

^a–d^ In each row, means with the same letter represented no significant differences. IL: interleukin; TNF-α: tumor necrosis factor-α; slgA: secretory IgA; TSLP: thymicstromal lymphopoietin; TGF-β: transforming growth factor-β. CON: the control group; OVA: mice orally challenged with OVA; OVA + LAVA: an OVA-challenged group that was oral gavaged with a low dose of 10 mg/kg bw AVA; OVA + HAVA: an OVA-challenged group that was oral gavaged with a high dose of 20 mg/kg bw AVA. Results are presented as means ± SDs (*n* = 9).

**Table 2:** Cytokines in the jejunum in experiment 2.

Variable	OVA	OVA + HAVA	OVA + HAVA + APO	OVA + APO	*p* Value
IL-4 (pg/mL)	36.2 ± 3.13 ^b^	23.23 ± 2.24 ^d^	31.52 ± 5.52 ^c^	42.41 ± 3.95 ^a^	<0.01
TNF-α (pg/mL)	519.3 ± 37.91 ^b^	378.6 ± 27.55 ^d^	440.5 ± 42.27 ^c^	628.6 ± 35.15 ^a^	<0.01
IL-10 (pg/mL)	29.33 ± 1.80 ^b^	35.88 ± 3.17 ^a^	34.89 ± 1.43 ^a^	23.58 ± 2.22 ^c^	<0.01
IL-25 (pg/mL)	55.22 ± 4.85 ^c^	80.37 ± 4.61 ^a^	69.18 ± 6.26 ^b^	47.19 ± 4.64 ^d^	<0.01
IL-33 (pg/mL)	123.5 ± 15.74 ^c^	181.6 ± 13.95 ^a^	163.3 ± 11.14 ^b^	106.9 ± 8.07 ^d^	<0.01
slgA (ng/mL)	2173 ± 154.0 ^c^	3048 ± 232.0 ^a^	2755 ± 128.8 ^b^	1901 ± 177.5 ^d^	<0.01
TSLP (pg/mL)	35.86 ± 3.38	37.58 ± 4.33	37.01 ± 4.16	35.03 ± 2.31	0.46
TGF-β (pg/mL)	406.9 ± 82.60 ^c^	690.4 ± 45.14 ^a^	592 ± 28.36 ^b^	303.2 ± 32.17 ^d^	<0.01

^a–d^ In each row, means with the same letter represented no significant differences. IL: interleukin; TNF-α: tumor necrosis factor-α; slgA: secretory IgA; TSLP: thymicstromal lymphopoietin; TGF-β: transforming growth factor-β. OVA: mice orally challenged with OVA; OVA + HAVA: an OVA challenged group that was oral gavaged with 20 mg/kg bw avenanthramide (AVA); OVA + HAVA + APO: the OVA + HAVA group coupled with apoptozole (APO); OVA + APO: the OVA group coupled with APO. Results are presented as means ± SDs (*n* = 9).
